# Beyond Access: A Qualitative Exploration of the Social and Behavioural Drivers of Routine Childhood Immunisation in the Solomon Islands

**DOI:** 10.3390/vaccines14100911

**Published:** 2026-10-19

**Authors:** Jake Turvey, Ben Sanderson, Jenniffer Anga, Rechelle Anga, Susan Mete, Chelsea Taylor, Stefanie Vaccher, Milena Dalton, Catherine King

**Affiliations:** 1 Burnet Institute, Melbourne, VIC 3004, Australia; jake.turvey@burnet.edu.au (J.T.); stefanie.vaccher@honorary.burnet.edu.au (S.V.); 2 Solomon Islands Ministry of Health and Medical Services, Honiara, Solomon Islands; 3 Independent Researcher, Honiara, Solomon Islands; 4 Sydney School of Public Health, Faculty of Medicine and Health, The University of Sydney, Sydney, NSW 2050, Australia; catherine.king@sydney.edu.au

**Keywords:** routine childhood immunisation, vaccine uptake, behavioural and social drivers (BeSD), Solomon Islands, Pacific island countries, community engagement, qualitative research

## Abstract

Background/Objectives: Gaps in routine childhood immunisation coverage persist in the Solomon Islands, with completion of the vaccination schedule varying considerably between remote and geographically isolated communities. While structural and logistical barriers to immunisation, such as vaccine supply constraints and inadequate cold chain infrastructure, are frequently documented in such settings, far less is known about the attitudes, norms, and relationships that shape caregiver and community engagement with immunisation services. This study aimed to apply the WHO Behavioural and Social Drivers of Vaccination (BeSD) framework to generate locally grounded evidence on the drivers of routine childhood immunisation in the Solomon Islands. Methods: An exploratory qualitative study was undertaken across three rural and peri-urban sites in Guadalcanal Province. Semi-structured in-depth interviews were conducted in Solomon Pijin with 16 participants, including caregivers, community influencers, healthcare workers, and immunisation program managers. Data collection was guided by the BeSD framework, with tools adapted through cognitive interviewing and pre-testing. Data were analysed using framework analysis, organised around the four BeSD domains. Results: Caregivers expressed broadly positive attitudes towards vaccination, though this coexisted alongside concerns about post-vaccination side effects and confusion about specific vaccine types. Social norms, community and religious leaders, and gendered caregiving expectations shaped engagement with immunisation services. Geographic isolation, financial cost, and facility-level constraints further impeded routine vaccination uptake. Conclusions: Addressing immunisation gaps in the Solomon Islands requires strategies that extend beyond structural and logistical improvements alone, integrating trusted caregiver communication, meaningful engagement with community and religious leaders, and sustained investment in health system capacity and outreach reliability to build lasting, community-driven improvements in routine childhood vaccination coverage.

## 1. Introduction

Despite sustained global efforts to expand routine childhood immunisation coverage, incomplete vaccination remains a major public health challenge [1]. In 2024, an estimated 20 million children worldwide were under-immunised against key vaccine-preventable diseases, of whom 14.3 million received no vaccines at all [1]. Unvaccinated and under-immunised children face significantly elevated risks of morbidity and mortality from vaccine-preventable diseases, while broader coverage gaps undermine community-level protection and increase the risk of outbreaks [1,2,3,4].

These vulnerabilities are particularly pronounced in the Pacific region, where recent outbreaks of measles and other vaccine-preventable diseases have highlighted the ongoing risk posed by suboptimal vaccination coverage [5,6,7]. In the Solomon Islands, routine childhood immunisation is characterised by relatively high first-dose coverage across most routine vaccines, but significant drop-out in subsequent doses [8]. Coverage of key childhood vaccines such as diphtheria–tetanus–pertussis (DTP) and measles–rubella (MR) has steadily increased in recent years, with first-dose rates reaching 91% and 78%, respectively, in 2024 [8]. However, completion of subsequent doses remains lower (DTP-3 87%; MR-2 66%), with dropout rates higher in more remote and isolated regions, including Malaita and Western Province [8,9]. In 2022, these provinces accounted for the largest proportions of under-immunised children nationwide, with 60% and 62% of children not receiving DTP-3, respectively [9]. Within these provinces, these under-immunised children are geographically concentrated, with 91% of under-immunised children in Malaita residing in just two districts, and 86% in Western Province residing across three districts [9].

Structural and logistical barriers, such as vaccine supply constraints and inadequate cold chain infrastructure, are widely recognised contributors to low vaccine uptake across the Pacific region [10]. However, these factors alone do not fully account for observed patterns of under-immunisation. A growing body of international evidence has highlighted the importance of social and behavioural determinants, including caregiver attitudes, perceptions of vaccine safety, social norms, and interpersonal dynamics, in shaping how families and communities engage with routine immunisation services [10,11,12]. Translating this evidence into effective immunisation programming requires a deeper understanding of how these determinants operate in specific local contexts, particularly in Pacific Island settings where evidence on community-level drivers of vaccine uptake remains limited [13].

To address this evidence gap, the World Health Organization (WHO) developed the Behavioural and Social Drivers of Vaccination (BeSD) framework in 2018, with formal guidance published in 2022 [14]. The framework and associated data collection tools provide a structured and systematic approach to identifying and measuring social and behavioural determinants of vaccine uptake at the individual and community level [14]. While the BeSD framework has been applied across diverse health settings internationally, to the best of our knowledge, this study is first to use the framework to understand the key factors influencing the uptake of routine childhood vaccinations in the Pacific region and other Small Island Developing States [15,16,17,18]. This study therefore aimed to use the BeSD framework to generate locally grounded evidence on the social and behavioural drivers of routine childhood immunisation in Guadalcanal Province, Solomon Islands, and identify strategies to strengthen immunisation policy and programming.

## 2. Materials and Methods

### 2.1. Study Setting

The Solomon Islands is a predominately Melanesian archipelago nation comprising nearly 1000 islands spanning six provinces, and covering approximately 28,500 km^2^ of the southwestern Pacific region [19]. With a population of over 800,000, approximately 80% of whom live in rural areas, many communities are small, geographically isolated, and accessible only by boat or on foot [20].

This study was conducted across three sites in Guadalcanal Province, including two highly isolated rural villages and one peri-urban community located on the outskirts of Honiara, the nation’s capital. Sites were selected based on prior analyses identifying communities with high proportions of under-immunised children, and in consultation with the Ministry of Health and Medical Services (MHMS) and local research officers. The study formed part of a broader program of research examining the adaptation and application of the BeSD tools across three Melanesian countries, including Vanuatu, Solomon Islands, and Papua New Guinea, with findings reported separately by country to allow for more in-depth, context-specific analysis.

### 2.2. Study Design and Tools

An exploratory qualitative design was employed to effectively capture the nuanced social and behavioural dynamics that characterise vaccination decision-making in remote island communities. Data collection was guided by the WHO BeSD framework and organised across its four primary domains: thinking and feeling, social processes, motivation, and practical issues [14]. UNICEF’s Journey to Health and Immunization (JTHI) framework was also used during pre-testing to provide complementary insights and help inform contextual adaptations to the BeSD study materials [21]. The JTHI framework examines the journey families take in accessing and completing immunisation services, capturing practical and systemic barriers alongside individual-level factors that shape engagement with vaccination programs [21].

Prior to data collection, three local research officers (R.A., S.M. and A.F.) were recruited through existing in-country research networks and provided with comprehensive online training covering qualitative research methods, application of the BeSD and JTHI tools, research ethics, data management, and cognitive interviewing techniques. Study materials were translated into Solomon Pijin by a local translation service and reviewed for accuracy by an independent MHMS employee.

Four cognitive interviews were subsequently conducted by A.F. to assess translation accuracy, question clarity, and cultural appropriateness of both the BeSD and JTHI frameworks and interview materials. Following minor refinements, R.A., S.M. along with study investigator J.A. pre-tested both tools with 14 individuals from two rural villages in Guadalcanal Province. Insights from pre-testing stage informed further adaptations to BeSD study tools, including refinements to the wording, structure, and the addition of supplementary questions to capture cultural and context-specific considerations. All subsequent data collection and analysis reported here were conducted using the adapted BeSD tools (File S1).

### 2.3. Participants and Recruitment

Participants were drawn from four key stakeholder groups, including caregivers of children under five years of age, community influencers such as religious and community leaders, frontline healthcare workers, and immunisation program managers. Caregivers and community influencers were recruited through convenience sampling, while healthcare workers and program managers were purposively recruited based on their roles in immunisation service delivery and program oversight. Informed consent was obtained from all participants prior to the commencement of research activities. Information describing the aims, confidentiality protections, voluntary nature of participation, and the right to withdraw was provided in both Solomon Pijin and English. For participants with low-literacy, study information was provided verbally by the local research officers with informed consent obtained verbally in the presence of an impartial witness, with the process documented in study records. Local research officers were available to answer questions prior to participation and throughout the interview process.

### 2.4. Data Collection and Analysis

In March 2024, semi-structured in-depth interviews were conducted using the adapted BeSD tools across three locations in Guadalcanal Province. Local research officers conducted interviews in Solomon Pijin, with audio recording undertaken for each session with participant consent. The adapted BeSD interview guides served as a general framework, with research officers able to explore topics raised by participants beyond the listed questions and prompts. Contextual observations were captured through field notes maintained across all data collection activities. Local research officers subsequently transcribed and translated audio recordings and field notes into English.

Framework analysis was selected as the analytical approach given its suitability for qualitative research organised around a predefined conceptual structure like the BeSD tools [22]. An initial codebook was developed based on the four BeSD domains, with supplementary codes added inductively as new themes emerged. Coding matrices were developed in Microsoft Excel 365 (Version 2402) and applied independently by two researchers (B.S., C.K.). Disagreements in coding were resolved through discussion and consensus review. Emerging themes were cross-checked against field notes and investigator observations. Local research officers and study investigators reviewed the findings throughout the analysis process to ensure contextual accuracy and relevance. Preliminary findings from the adaptation and pre-testing phase were presented to MHMS in September 2023, and a summary of the full study findings was provided to the Ministry, study participants, and partner organisations at the conclusion of the study.

This project received approval from the Solomon Islands Health Research and Ethics Review Board (HRE018/23) and The Alfred Hospital Ethics Committee (Project No. 325/23).

## 3. Results

### 3.1. Demographics

In total, 16 participants took part in this study, of whom 11 (69%) were female, with a mean age of 42 years. Participants were drawn from four stakeholder groups, including caregivers of children under five (*n* = 6), community influencers (*n* = 4), frontline healthcare workers (*n* = 4), and immunisation program managers (*n* = 2). All caregivers were female and all community influencers were male, reflecting prevailing gender norms across the sampled locations. Further participant characteristics are summarised in Table 1.

### 3.2. Qualitative Results

**Figure 1:**
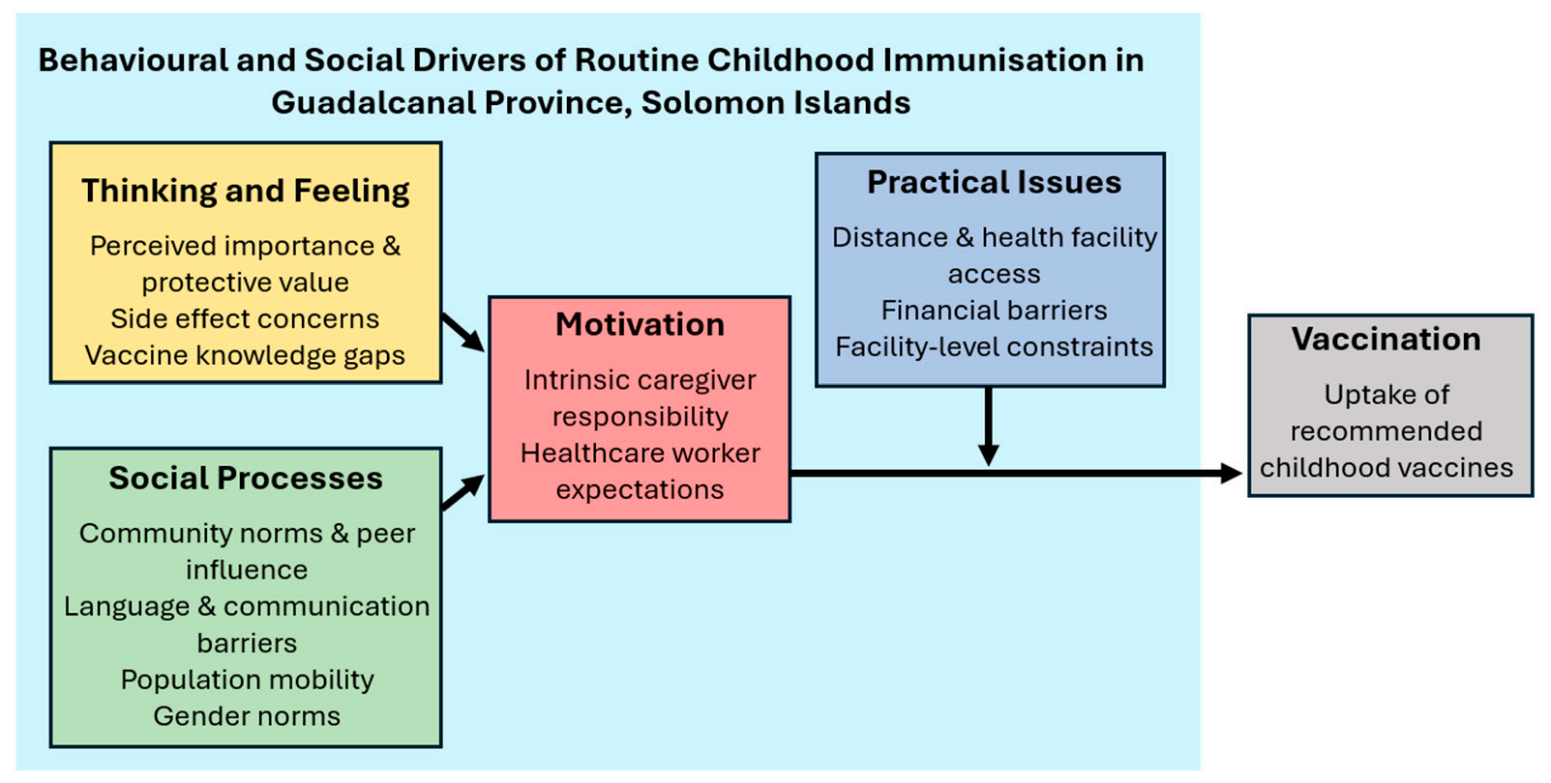
The Behavioural and Social Drivers of Routine Childhood Immunisation in Guadalcanal Province, Solomon Islands, mapped to the BeSD Framework [14].

Qualitative findings are drawn from in-depth interviews conducted across all four participant groups. Key findings are presented below and have been mapped across the four BeSD framework domains (Figure 1) [14]. All participant identifiers have been anonymised to preserve confidentiality, with quotes attributed by participant group and a unique participant number (e.g., Caregiver, P01).

#### 3.2.1. Thinking and Feeling

##### **Perceived Importance and Protective Value** 

Caregivers across both peri-urban and rural settings expressed strong support for routine childhood vaccination, describing it as important for protecting their children’s health and preventing disease. Notably, these expectations were often framed in broad terms, with caregivers describing vaccines as protection against illness more generally, rather than specific vaccine-preventable diseases:

*“It is a good thing because it contributes to the health of the child … I think it is important to help the child to not get sick.”*

(Caregiver, P01)

*“[vaccination] is important, because nowadays there are a lot of diseases affecting a lot of the children … It will protect the babies from getting diseases.”*

(Caregiver, P06)

Many caregivers also described positive feelings following their child’s immunisation, expressing satisfaction and confidence in their decision to vaccinate:

*“For us when our baby gets the vaccine, we are very happy about it, I feel happy that my baby gets the vaccine.”*

(Caregiver, P06)

*“We feel that it [children’s vaccine] a good thing … from my opinion I think everything about the vaccine is good.”*

(Caregiver, P05)

##### **Side Effect Concerns** 

While caregivers expressed broadly positive views towards vaccination, concerns about post-vaccination side effects emerged as a common source of uncertainty. Caregivers commonly described distress at observing their children experience pain and prolonged crying following vaccination, reactions that generated anxiety and apprehension around the vaccination experience:

*“Sometimes it’s not okay for the baby, because the baby will be in pain and crying a lot.”*

(Caregiver, P04)

*“I always worry about my child crying a lot and having pains after getting the vaccine.”*

(Caregiver, P06)

Community influencers also identified fear of adverse reactions as a widely observed barrier to schedule completion within their communities, with some caregivers reportedly avoiding subsequent doses as a result:

*“Some [caregivers] don’t get their children to get the vaccine because of the side effects. That is why they don’t go back for their next vaccine.”*

(Community Influencer, P12)

*“Most mothers believe that their babies will get sick if they get the vaccine.”*

(Community Influencer, P14)

Caregivers also described the practical burden of caring for an unwell child as disruptive to daily responsibilities, reinforcing their reluctance to return for subsequent vaccination visits:

*“When the baby is crying, I feel tired because of non-stop crying, and it makes me not want my child to get the vaccine.”*

(Caregiver, P04)

*“The baby will cry a lot, which prevents us from completing some of our other tasks that need to be done.”*

(Caregiver, P02)

##### **Vaccine Knowledge Gaps** 

Gaps in vaccine knowledge influenced how caregivers engaged with routine immunisation services. Healthcare workers commonly reported confusion between routine childhood vaccines and the COVID-19 vaccine as a driver of refusal, with some caregivers declining routine doses out of fear that their child might inadvertently receive a COVID-19 vaccine, despite children under five being ineligible for COVID-19 vaccination in the Solomon Islands. This concern persisted even after health workers attempted to clarify the distinction:

*“Last year I notice that some [caregivers] get confused with the COVID-19 vaccine. They think the nurses might give them the COVID-19 vaccine instead of the children’s vaccine, thus they refuse to get the vaccines. Even though we explain to them they just refuse to get their routine vaccine.”*

(Healthcare Worker, P08)

*“One reason they give when we go to the communities is the COVID-19 vaccine. They don’t want their children to be immunised with the COVID-19 vaccine. They say because a lot [of] people that get the COVID-19 vaccine have experienced side effects like knee pain.”*

(Healthcare Worker, P09)

More broadly, healthcare workers described limited understanding among caregivers of how vaccines work and which diseases they protect against. These knowledge gaps created practical difficulties in schedule navigation, with some caregivers misinterpreting vaccine types or losing track of appointments, contributing to missed doses and reduced engagement with immunisation services over time:

*“When we got out to do campaigns and give out the measles vaccines and other vitamins, some [caregivers] think it is the 1-year vaccine, thus they won’t come to the clinic anymore. They do get confused with the schedule and forget about their routine vaccines.”*

(Healthcare Worker, P09)

*“Some mothers just don’t understand the information about the vaccine, that is why they don’t come to the clinic.”*

(Healthcare Worker, P10)

#### 3.2.2. Social Processes

##### **Community Norms and Peer Influence** 

Caregivers’ perceptions of vaccination were strongly shaped by the behaviours and attitudes of their peers, family, and community members. In peri-urban settings, caregivers generally described vaccination as a common and accepted practice among their peers, though hesitancy was not entirely absent, with some parents reportedly delaying vaccination due to fear or competing priorities:

*“A lot of the women I know bring their children to get the vaccine, but some mothers say that they are afraid of the vaccine, and some say that they are just being lazy.”*

(Caregiver, P01)

In rural settings, caregivers reported that vaccination was less consistently practiced among their peers, yet described meaningful informal support networks through which parents helped one another track vaccination dates and reinforced the importance of immunisation:

*“Most of the parents I know don’t take their children for vaccination… but sometimes if we know the vaccination date of other children, we remind the other parents on the date. We also talk to them about the importance of the vaccine.”*

(Caregiver, P06)

Community leaders, including village chiefs and church representatives, were also described as playing an active role in reinforcing vaccination norms in rural areas, working alongside caregivers to encourage attendance at vaccination sessions:

*“… for us, the community leaders like the chief and church leaders, we all work together and remind everyone they must bring their children to the clinic for vaccination.”*

(Caregiver, P05)

Community influencers further served as key intermediaries between health services and the broader community, disseminating information about upcoming outreach visits through village meetings and church services. In rural communities, where advance notice was essential for families to plan around work and travel commitments, this communication role was particularly consequential as its breakdown was described as directly contributing to missed vaccination opportunities:

*“Health workers notify us before they come, so we can tell people, otherwise they are out working and miss the vaccines.”*

(Community Influencer, P11)

Despite occupying this central role, community influencers described feeling ill-equipped to deliver accurate vaccine information, identifying a gap between their motivation to support immunisation and their capacity to address caregiver concerns effectively:

*“I advise other people to go to the clinic, it’s true [vaccination] is a good thing. But I don’t have the knowledge and training to do so.”*

(Community Influencer, P11)

Religious beliefs represented a further dimension of community influence, with healthcare workers identifying doctrinal opposition to vaccination among members of certain minority faith communities as a localised, yet distinct barrier to immunisation in some settings:

*“[Specific denomination] don’t allow their children to get the vaccine, even getting treatment for their children they don’t allow that as well. Even when they deliver their baby, they don’t allow us to give BCG [Bacillus Calmette–Guérin vaccine] or HepB [Hepatitis B vaccine] because of their religious beliefs.”*

(Healthcare Worker, P07)

*“Some reasons people give [for vaccine refusal] is due to their church beliefs and their doctrines which don’t allow their children to get the vaccine or vaccinated.”*

(Healthcare Worker, P10)

##### **Language and Communication Barriers** 

Language barriers between health workers and caregivers emerged as a meaningful constraint on immunisation engagement in rural settings. Nurses posted from other provinces often did not speak local languages, creating confusion and hesitancy among caregivers who were unable to understand or communicate with clinic staff:

*“Most of the people don’t speak Pijin, so they don’t bring their children to the clinic because they are scared … they don’t come because they don’t know and don’t understand what the nurses are saying to them.”*

(Caregiver, P05)

*“The other thing is language barrier, because most of the people don’t speak Pijin and most of the nurses are from other provinces, so they don’t speak our language.”*

(Community Influencer, P14)

These difficulties extended beyond verbal communication. Limited health literacy around vaccine card use meant that some caregivers attended clinics on incorrect dates and were turned away, an experience that appeared to erode trust and discourage continued engagement with immunisation services:

*“A few of us knows how to check the card for the date, but for some who don’t know, they don’t check at all. Most times they come to the clinic the nurse turned them back because it is not their vaccination day.”*

(Caregiver, P05)

##### **Population Mobility** 

Program managers identified population mobility as another contributing factor to missed vaccinations, with families moving for seasonal employment, election campaigns, or other work commitments, frequently losing continuity with local health services as a result:

*“These people move around with campaigns for the upcoming election, so we have missed a couple of families when we came in and cross-checked with the register.”*

(Program Manager, P16)

Program managers suggested that targeted strategies, such as liaising with local community leaders or tracking mobile families, could help ensure these children receive timely vaccinations, while village health committees could potentially help with broader health promotion activities in rural areas.

##### **Gender Norms** 

Across study sites, mothers were consistently identified as the primary managers of childhood vaccination across participant groups, reflecting broader social expectations around caregiving responsibilities. The commonly held expectation that the responsibility for vaccination fell to female caregivers carried practical implications, as limited paternal involvement or household support could create vulnerabilities in vaccination continuity, particularly when mothers faced competing demands or required assistance to access services:

*“Every time we notice that only the mothers bring their children to the clinic.”*

(Healthcare Worker, P09)

While fathers occasionally accompanied their wives to appointments, caregiving support was predominantly drawn from female kinship networks, with sisters and grandmothers helping to manage other children at home or accompanying mothers to the clinic:

*“Sometimes my husband will follow us to the clinic and leave us there and he will go to work … sometimes my sister or my mother takes care of my other child.”*

(Caregiver, P01)

#### 3.2.3. Motivation

##### **Intrinsic Caregiver Responsibility** 

Caregivers described a mix of intrinsic and externally reinforced motivations for vaccination attendance. For many, the decision to vaccinate was driven by a strong personal sense of parental responsibility, with caregivers articulating vaccination as a non-negotiable commitment to their child’s health regardless of competing demands:

*“Our children, all of them had to get the vaccine, even though we might be tired or are being lazy, we have to go to the clinic.”*

(Caregiver, P06)

##### **Healthcare Worker Expectations** 

Healthcare workers also played an active role in sustaining motivation, with nurses regularly prompting attendance by writing appointment dates in children’s medical books and reinforcing the importance of completing the vaccination schedule. For some caregivers, this guidance extended beyond encouragement, generating a sense of social obligation that itself became a driver of attendance:

*“The nurse strongly advised us to bring our children to get the vaccine.”*

(Caregiver, P05)

*“If I miss [the appointment] the nurses will be frustrated with me.”*

(Caregiver, P04)

#### 3.2.4. Practical Issues

##### **Distance and Health Facility Access** 

Physical distance and difficulty accessing health facilities constituted a significant barrier to vaccination across all participant groups, with these challenges being especially pronounced at rural study sites. Caregivers described arduous journeys requiring long walks or boat travel, frequently disrupted by weather conditions that rendered routes impassable:

*“If there is no form of transport, walking will be our only way to get to the clinic … when there is rain, and the river gets flooded, then we can’t come to the clinic.”*

(Caregiver, P05)

For some caregivers in remote communities, accessing vaccination services required overnight stays in neighbouring villages, with the additional burden of carrying supplies and other children compounding the physical and logistical demands of the journey:

*“… if there is no boat, then we have to sleep at that village and then we walk to the clinic […] we carry our bags, carry the child and follow the road which is a long way. Sometimes it is very tiring.”*

(Caregiver, P04)

Caregivers and community influencers both suggested that the establishment of more local health facilities was a potential solution, noting that village-based clinics would not only reduce travel demands but also address the apprehension some caregivers experienced when navigating unfamiliar settings:

*“If there is a clinic available in my community it will really help a lot and a lot of the children in my community will get the vaccine.”*

(Community Influencer, P14)

*“Most parents are scared of coming to a bigger place like this, so by having a clinic will help parents come to the clinic.”*

(Caregiver, P05)

From a service delivery perspective, program managers acknowledged the severity of these practical barriers, whilst also describing the considerable operational challenges they themselves experienced when extending outreach to dispersed and isolated communities:

*“Transport and weather are two things that stop [caregivers] from coming in for vaccination.”*

(Program Manager, P15)

*“A population of no more than a thousand people, very small, living in scattered set ups. They don’t live in one big community. So, it’s like you have to climb hills to go to visit 20 people. They are not living together so it’s difficult […] no nurse wants to go up there, it’s very isolated.”*

(Program Manager, P16)

Satellite clinic visits were identified by program managers as a partial solution, though their implementation was similarly constrained by resource availability, with transport and fuel shortages frequently preventing planned visits from proceeding:

*“We haven’t done [satellite visits] this year. In our previous workplan we would do satellites once a week … if we have transport and fuel we usually go out to the communities, but if there is no transport then we just stay here at the clinic.”*

(Program Manager, P16)

##### **Financial Barriers** 

The financial costs associated with accessing vaccination services represented a significant and compounding barrier for caregivers, particularly those in remote areas. Transport costs were frequently described as the primary financial burden, with some caregivers foregoing meals during clinic journeys to manage expenses:

*“If we have money to spend then we will pay [for food], if not then we will just be hungry and wait till we go home.”*

(Caregiver, P06)

*“Most parents live far and they need money for transport in order to get to the clinic.”*

(Healthcare Worker, P07)

The financial burden of accessing services was not limited to caregivers. Health workers described facing similar constraints in funding the transport and fuel costs required to conduct outreach activities, pointing to a shared financial vulnerability across both the demand and supply sides of immunisation delivery:

*“Money is the primary factor that influences how quickly people can access healthcare services. We must take into account the transportation costs required to reach the clinic. Often, they need to take multiple buses, and sometimes their funds are insufficient […] many of the people in the community don’t have money and you know it’s hard carrying your child and walking in the hot sun. They have to struggle for ways to make money in order to afford going to the clinic.”*

(Community Influencer, P11)

##### **Facility-Level Constraints** 

At most health facilities serving the studied communities, childhood vaccination was delivered on designated child welfare days that also encompassed a range of other child health services. The resulting demand on these sessions was described as generating consistently long wait times, with some caregivers unable to be seen despite attending on the correct day:

*“Sometimes when the women arrive at the clinic, they may end up 70th [in] line if they are a bit late … sometimes when they went to the clinic and got hungry, they have to return with their children even if they don’t see the nurses because of the waiting line.”*

(Community Influencer, P11)

Vaccine stockouts were also identified as a recurring constraint, with supply chain challenges leaving some facilities unable to provide the full range of scheduled vaccinations needed by attendees. At the time of data collection, one facility had only a single vaccine type available, with all other routine vaccines out of stock:

*“The only problem that I have currently is with the stock access. We have been sending orders through, but I think the issue is the stock management … so when we place orders, they don’t come in time.”*

(Program Manager, P16)

Record-keeping was identified as another facility-level challenge, with healthcare workers noting that documentation demands exceeded available resources during busy clinic days. Program managers suggested strengthening data recording systems as a priority, recognising its importance for identifying coverage gaps and supporting catch-up vaccination efforts:

*“The documentation part is what we really need to strengthen, because if all these books and papers are missing then it will be a problem. We need a computer or anything that will make it easier for recording for the staff to work on […] Proper recording will help us to know what problems are there and will help us to do catch ups.”*

(Program Manager, P15)

## 4. Discussion

This study found a broadly supportive environment for routine childhood immunisation in the Solomon Islands, with caregivers consistently describing vaccination as important. Yet this support coexisted alongside persistent concerns, including uncertainty about vaccine effects, confusion regarding specific vaccines, and gaps in understanding of the immunisation schedule. This pattern is consistent with existing evidence from the Solomon Islands, including national survey data in which 96% of respondents reported willingness to vaccinate with any MHMS-endorsed vaccine, while 42% also held strong concerns about vaccine effects [23,24,25]. This tension highlights the complexity underlying caregiver engagement with immunisation services in the Solomon Islands, reinforcing the need for immunisation programs that not only improve vaccine access and service delivery, but also strengthen vaccine confidence and improve caregivers’ understanding of routine childhood immunisation.

Caregiver confidence in vaccination was particularly undermined by confusion between routine childhood vaccines and the COVID-19 vaccine, with healthcare workers reporting that some caregivers refused routine doses out of fear of inadvertently receiving a COVID-19 vaccine. This concern persisted despite active clarification from healthcare workers, illustrating how perceived risk can take precedence over corrective information in shaping caregiver behaviours. Previous research has similarly demonstrated that fear can be a powerful driver of vaccine hesitancy, highlighting the broader influence of emotion on vaccination decision-making [26]. Concerns about post-vaccination side effects operated through a related but distinct mechanism, with community influencers identifying fear of adverse reactions as a barrier to schedule completion, and some caregivers reportedly avoiding subsequent doses as a result. This suggests that direct, lived experience of a child’s discomfort can shape caregiver engagement independently of broader attitudes towards vaccination. Strengthening the capacity of local healthcare workers and community influencers to engage with caregivers’ underlying fears and concerns in a language and context they understand will be important for improving vaccine confidence in the Solomon Islands. Interpersonal communication training equips health workers to respond to these emotional concerns directly, and has been shown to contribute significantly to improved vaccination outcomes in comparable settings, representing a promising approach for strengthening caregiver trust and confidence in routine immunisation services [27,28,29].

The prominent role of social networks and community figures in shaping caregiver vaccination behaviour observed in this study is consistent with a well-established body of evidence demonstrating that immunisation decisions are closely influenced by the norms, experiences, and recommendations shared within caregivers’ immediate social environments [30,31]. Peer relationships among mothers and female relatives in particular were described as important informal channels for health information and mutual reinforcement of vaccination norms, reflecting patterns also documented across similar settings where formal health system contact is limited or irregular [30]. Community leaders were similarly identified as active supporters of vaccination in their local areas, yet many described feeling underprepared to fulfil this role effectively, citing limited technical knowledge and insufficient interpersonal communication skills as barriers to addressing caregiver concerns. This gap between the central position community leaders occupy in local health communication and their capacity to deliver accurate vaccine information represents both a challenge and opportunity for immunisation programming in the Solomon Islands. Investments in the capacity of trusted community intermediaries, including through structured Vaccine Champion-style programs [28] that engage community leaders, women’s groups, and peer networks, could meaningfully extend the reach and credibility of immunisation messaging in settings where formal health system contact alone is insufficient [32].

Religious leaders in particular can serve as critical trust-builders within communities, communicating health messages in ways that align with local cultural and spiritual understandings [33]. Their engagement in vaccination programs during the COVID-19 pandemic was widely shown to effectively support public health efforts by influencing health behaviours, and framing vaccination as both a moral duty and collective responsibility [33]. However, religious beliefs can also constrain vaccine uptake, as evidenced by study participants who reported that certain faiths or doctrinal interpretations actively restricted vaccination in their communities. National-level survey data reinforces this tension, with only 44% of Solomon Islands respondents believing vaccination aligns with their religious values [34]. These findings highlight the dual role of religion in shaping vaccine health behaviours, and underscore the importance of engaging religious leaders thoughtfully, leveraging their trusted position to promote vaccination while remaining attentive to concerns that may contribute to hesitancy in some communities [33].

Practical and logistical barriers were described as a persistent and significant constraint on timely immunisation across participant groups, echoing findings from across the Pacific and other low- and middle-income settings [35,36,37,38]. Geographic isolation, transport constraints, and the financial burden of accessing services were major themes in this study, reflecting a broader regional picture in which physical and economic barriers continue to concentrate under-immunisation among the most remote and marginalised communities [36,39]. Limitations in outreach services were a particularly prominent finding in this study, corroborated by a recent WHO-led review of immunisation services in the Solomon Islands, which found that while outreach activities cover approximately 40% of the population, many sessions do not proceed as planned due to persistent funding, fuel, and workforce shortages [39]. Addressing these gaps in the Pacific context requires not only sustained investment in transport infrastructure and workforce capacity, but deliberate attention to the logistical realities of delivering services across dispersed island communities, including reliable fuel access, community-level outreach coordination, and contingency planning for weather-related disruptions [39]. The feasibility of such investments, however, will depend on the broader funding landscape for immunisation in the Pacific, where shifts in donor support and increasing pressure on domestic health budgets present ongoing challenges to program sustainability [39].

Finally, health system constraints such as vaccine stockouts, health worker shortages, and documentation gaps undermined service reliability and compounded the barriers caregivers faced in accessing immunisation. These findings align with the broader assessment of the Solomon Islands immunisation program outlined in a recent WHO-led review, which similarly identified cold chain management, workforce capacity, and supportive supervision as priority areas for improvement [39]. Addressing these foundational health system capacities remains essential to improving both facility-based and outreach vaccination delivery. Evidence from within the Solomon Islands illustrates the potential of targeted system-strengthening approaches. A 2022 intervention providing microplanning training, supportive supervision, and improved data monitoring in line with the WHO’s Reaching Every District framework resulted in reported coverage increases of 1.5 to 3 times higher in two low-performing Solomon Islands provinces over nine months [9,40]. This finding, alongside a broader body of evidence from comparable resource-limited settings, demonstrates that sustained technical and system-level investment can meaningfully improve immunisation program performance in the Solomon Islands [41,42].

### 4.1. Implications for Immunisation Programming

The findings of this study hold several implications for immunisation policy and programming in the Solomon Islands. A range of potential strategies to strengthen routine childhood immunisation emerged both from participant responses and through the discussion of these findings against the wider evidence. These strategies are summarised in Table 2.

### 4.2. Strengths and Limitations

This study represents the first application of the WHO BeSD framework to examine routine childhood immunisation in the Solomon Islands and the wider Pacific region, and is among only a few BeSD studies conducted in the Pacific to date, helping to address a critical evidence gap. The findings provide practical insights to guide routine immunisation program strengthening in the Solomon Islands and other similar regional settings. Data collection was led by local research officers working in Solomon Pijin and was conducted in close collaboration with the Solomon Islands Ministry of Health and Medical Services, enabling in-depth community engagement and ensuring that participant perspectives were captured in contextually appropriate and linguistically accessible ways. Additionally, this study drew on a diverse range of participant groups, including caregivers, healthcare workers, program managers, and community and religious leaders across both peri-urban and rural settings, providing a multifaceted view of the social and behavioural drivers of immunisation in the Solomon Islands. Finally, the iterative adaptation and refinement of the BeSD tools through cognitive testing and pre-testing resulted in instruments more closely grounded in local context, language, and lived experiences, allowing a more authentic capture of participant perspectives and decision-making processes.

While the exploratory study design was purposely selected to prioritise analytical and contextual depth over population-level inference in an understudied setting, the small sample size nevertheless limits the generalisability of the findings across the Solomon Islands and other Pacific island communities. Furthermore, despite considerable effort to recruit caregivers of under-immunised or unvaccinated children, all participating caregivers reported their children as vaccinated, meaning the study may underrepresent the barriers and decision-making processes specific to households where children have missed vaccination. Male caregivers were also not represented, limiting insights into their role and influence in immunisation decisions. Future research should seek to extend these findings through targeted engagement with more communities and underrepresented groups, including unvaccinated households and male caregivers.

## 5. Conclusions

Addressing persistent immunisation coverage gaps in the Solomon Islands requires a nuanced understanding of the multiple factors shaping caregiver engagement with vaccination services, extending beyond structural barriers to address the social and behavioural dynamics operating at the community level. This study found a broadly supportive environment for vaccination amongst participants, with caregivers consistently expressing positive attitudes toward vaccination and strong community norms reinforcing immunisation. Yet this support coexisted alongside a range of attitudinal, social, and practical concerns that continue to shape engagement with routine immunisation services.

The adapted BeSD tools developed through this study offer a context-appropriate approach for examining these concerns and the drivers that underpin them. Wider application of these tools with national, provincial, and regional health partners, particularly in communities with high proportions of zero-dose and under-immunised children, would build a deeper understanding of these drivers and how they differ across diverse Pacific Island settings. Such insights would further support targeted, locally tailored programming that promotes more equitable and sustained uptake of routine childhood immunisation across the region.

## Figures and Tables

**Table 1:** Participant demographics.

Participant Group	Number of Participants (*n*)	Female *n*, (%)	Mean Age (Years)
Caregivers	6	6 (100)	32
Community Influencers	4	0 (0)	45
Healthcare Workers	4	4 (100)	49
Program Managers	2	1 (50)	54
Total	16	11 (69)	42

**Table 2:** Participant-identified and evidence-based strategies to strengthen routine childhood immunisation in the Solomon Islands.

BeSD Domain	Focus Area	Participant-Identified Strategies	Evidence-Based Strategies
Thinking and feeling	Vaccine information and side effects	N/A	Interpersonal communication training for local healthcare workers to respond to caregiver fears, including concerns about side effects and misinformation [27,28,29]
Social processes	Community engagement and communication	Advance notice of outreach visits from health workers to community leaders Liaising with community leaders to locate and track mobile families Village health committees to support health promotion	Engaging religious leaders as trusted messengers, while remaining attentive to doctrinal concerns [33] Drawing on peer networks and women’s groups to reinforce vaccination norms [30,31,32] Structured Vaccine Champion-style programs to train community leaders and healthcare workers in vaccine knowledge and communication [28,32]
	Training and capacity	Strengthening community influencers’ vaccine knowledge	Structured Vaccine Champion-style programs to train local community leaders and healthcare workers in vaccine knowledge and communication [28,32]
Motivation	Healthcare worker reinforcement	Healthcare workers prompting attendance by recording appointment dates and reinforcing schedule completion	N/A
Practical issues	Access and outreach	Village-based clinics in remote communities Satellite clinic visits supported by reliable transport and fuel	Sustained investment in transport infrastructure and workforce capacity [39] Reliable fuel access, community-level outreach coordination, and contingency planning for weather-related disruptions [39]
	Facility services and supply	Strengthened record-keeping to identify coverage gaps and support catch-up vaccination	Strengthened cold chain and vaccine stock management, local workforce capacity, and facility record-keeping [39] Microplanning, supportive supervision, and improved data monitoring [9,40]

## Data Availability

Due to ethical restrictions, data underlying the results presented in this study are not publicly available. De-identified data may be made available upon request from the corresponding author, subject to institutional and ethics approval.

## Supplementary Materials

The following supporting information can be downloaded at https://www.mdpi.com/article/10.3390/vaccines14100911/s1. File S1: Solomon Islands BeSD Participant Interview Guides.

## Abbreviations

The following abbreviations are used in this manuscript:

BCG	Bacillus Calmette–Guérin
BeSD	Behavioural and Social Drivers of Vaccination
COVID-19	Coronavirus Disease 2019
DTP	Diphtheria–tetanus–pertussis
HepB	Hepatitis B
JTHI	Journey to Health and Immunization
MHMS	Ministry of Health and Medical Services
MR	Measles–rubella
UNICEF	United Nations International Children’s Emergency Fund
WHO	World Health Organization
