# Supplementary material for: Beyond Access: A Qualitative Exploration of the Social and Behavioural Drivers of Routine Childhood Immunisation in the Solomon Islands
Source: Vaccines (Basel). 2026 Oct 19;14(10):911. doi: 10.3390/vaccines14100911 (PMC13709442; doi:10.3390/vaccines14100911)
Supplement: Supplementary file 1 [file vaccines-14-00911-s001.zip › File S1 Solomon Island BeSD Participant Interview Guides.pdf]

Adapted BeSD Tool - Qualitative Questions for Caregivers

Solomon Islands - March 2024

<table>
<tr><th>Question</th></tr>
<tr><td>Thinking back to the first time you had your child vaccinated, tell me why you decided that you would go ahead with it. [If first vaccine was administered at birth, ask about the first time they took their child back for their next set of scheduled vaccines.]

Probe:
Did anyone suggest it?
Who decided that you should take your child to be vaccinated?
Who usually takes your child(ren) to have their vaccines?</td></tr>
<tr><td>Do you talk about vaccination with anyone else?

Probe:
Who do you talk to?
What do they say?
Do other parents you know vaccinate their children?</td></tr>
<tr><td>Thinking back to the first time you took your child to be vaccinated, tell me how you knew it was time to do so?

Probe:
What kind of reminders do you use</td></tr>
<tr><td>Thinking about vaccination day for your child, tell me about what happens before you arrive at the place where your child gets the vaccine. Start with before you leave home.

Probe:
What do you need to do to prepare before you leave home?
How do you travel to the vaccination place?
Does anyone help you in any other way so you can get your child to the vaccination place?</td></tr>
<tr><td>When you take your child to be vaccinated, do you need to take time away from other duties, such as work or responsibilities at the home?

Before you answer, what do you think this question is trying to ask?

Record Response:</td></tr>
<tr><td>Once you arrive at the vaccination place, tell me what happens next.

Probe:
Who do you talk to when you get there?
What happens in the waiting room or queue?
Do you need to pay a fee?
Are other health checks done while you’re there?</td></tr>
</table>

**What happens when it's your child's turn to get the vaccine?**<br><br>**Probe:**<br>• What happens first? [Probe for each step until the vaccination is completed.]<br>• What do the health workers talk to you about while you're there?<br>• How do you feel when you talk with them?
**After your child has had the vaccine, tell me what happens next.**<br><br>**Probe:**<br>• What happens when you leave the vaccination place?<br>• How do you travel home?
**What do you like about what happens on vaccination day?**<br><br>**Probe:**<br>• Ask about each step described by the caregiver in the question above.<br>• [If there is something identified that they like] Why do you like it?
**What don't you like about what happens on vaccination day?**<br><br>**Probe:**<br>• [If the response is "nothing", list the steps in the process they describe and ask whether there is anything they don't like about them individually.]<br>• Is there anything you find difficult? Why do you find it difficult?<br>• What happens after you arrive home?
**Tell me how you feel about childhood vaccination?**<br><br>**Probe:**<br>• Why do you feel this way?<br>• Do you think it's a good thing? Why?<br>• Do you think it's important? Why?<br>• Is there anything you feel isn't good about vaccination? Can you tell me more about it?
**How do you feel when your child is vaccinated?**<br><br>**Probe:**<br>• Do you think it's good for your child? Why?<br>• Is there anything that worries you? Why does it worry you?
**What do you think would help parents in your community get their child vaccinated?"**<br><br>Before you answer, what do you think this question is trying to ask?<br><br>**Record Response:**
**Is there anything else you'd like to say?**

Adapted BeSD Tool - Qualitative Questions for Community Leaders
Solomon Islands - March 2024

**Section 1: Confirming consent and eligibility**		
	Document Date of interview	DATE
	Confirm consent has been provided	YES<br>NO
	Confirm participant meets all eligibility criteria:<br>*Is the community member in a position of leadership or position of influence within the community*	YES<br>NO

**Record Study Identification Number**	
Location of Interview	*List name of*<br>VILLAGE<br>WARD/ZONE<br>DISTRICT
Hello, I am [INTERVIEWER'S NAME] with [INSTITUTION OR ORGANIZATION NAME].<br>We are interviewing people to help improve vaccination services in [NAME OF COUNTRY]. The interview is expected to take __ minutes.<br>Your participation is completely voluntary and anonymous. The answers you give will be completely confidential. If you do not want to answer a question or wish to stop the interview, just let me know.<br>Would you be willing to take part in an interview with me?<br>[If audio recording the interview]<br>Would you be happy for me to record our conversation?	
What is your gender?	Woman<br>Man<br>Other<br>Prefer not to say?
How old is the participant in years?	Less than 20 years<br>20 – 29 years<br>30 – 39 years<br>40 – 49 years<br>50 – 59 years<br>60 years or above
What is the highest level of education you have completed?	None<br>Primary (grade 8 or less)<br>High (grade 9, or 10)<br>Secondary (grade 11 or 12)<br>Tertiary college<br>Other (please specify)
What is your position of leadership within the community? i.e.<br>• religious leader<br>• community health leader	Free text response

• community elder<br>• other?	

Question
**<u>Tell me a little about yourself and your role here in the community</u>**<br><br>**Probe:**<br>• To what extent does your work involve immunization?<br>• Can you tell me more about that?<br>• Who do you work with to do that work
**<u>Can you take me through the process you follow when you work in a community?</u>**<br><br>**Probe:**<br>[Note: This probe is for participants who work with families.]<br>• When you visit a family:<br>• What do you talk about?<br>• What information can you not leave without saying?<br>• Do you follow up with the families afterwards? How do you do that?<br><br>[Note: This question is for participants who work with other people and organizations; use as appropriate for the participant.]<br>• How do you help the front-line health workers in working with families?<br>• How do you help with routine immunization?
**<u>What do you find works in helping families stay up to date with their children's immunizations?</u>**<br>**Probe:**<br>• What helps them not miss doses or appointments?<br>[Note: This is to probe for practical issues.]<br>• What helps those who are hesitant about getting their children vaccinated?
**<u>What makes it difficult for families stay up to date with immunization?</u>**<br>**Probe:**<br>• Can you give some examples of reasons people give when their child has fallen behind the vaccination schedule?<br>• Can you give some examples of reasons that people give for refusing vaccines for their children?<br>• Are you able to overcome these challenges? How?
**<u>If you had the chance, what would you do to improve immunization services in your area?</u>**

Adapted BeSD Tool - Qualitative Questions for Health Care Workers
Solomon Islands - March 2024

**Section 1: Confirming consent and eligibility**	
Document Date of interview	DATE
Confirm consent has been provided	YES<br>NO
Confirm participant meets eligibility criteria:<br>*Healthcare worker/vaccinator/clinician/nurse*	YES<br>NO

**Record Study Identification Number**	
Location of Interview	*List name of*<br>VILLAGE<br>WARD/ZONE<br>DISTRICT
Hello, I am [INTERVIEWER'S NAME] with [INSTITUTION OR ORGANIZATION NAME].<br>We are interviewing people to help improve vaccination services in [NAME OF COUNTRY]. The interview is expected to take __ minutes.<br>Your participation is completely voluntary and anonymous. The answers you give will be completely confidential. If you do not want to answer a question or wish to stop the interview, just let me know. Would you be willing to take part in an interview with me?<br>[If audio recording the interview]<br>Would you be happy for me to record our conversation?	
What is your gender?	Woman<br>Man<br>Other<br>Prefer not to say?
How old is the participant in years?	Less than 20 years<br>20 – 29 years<br>30 – 39 years<br>40 – 49 years<br>50 – 59 years<br>60 years or above
What is the highest level of education you have completed?	None<br>Primary (grade 8 or less)<br>High (grade 9, or 10)<br>Secondary (grade 11 or 12)<br>Tertiary college<br>Other (please specify)
Qualification	Doctor<br>Health Extension Officer<br>Nurse<br>Community Health Worker<br>Midwife or nurse<br>Other (please specify)

How long have you been working in this profession?	<5 years<br>5-<10 years<br>≥10 years

Question
**Tell me a little about yourself and what you do.**<br><br>**Probe**:<br>• What are you responsible for?<br>• How many days do you work in this role?<br>• Where do you perform your duties?
**To what extent does your role involve immunization?**<br><br>**Probe**:<br>• What parts of your job involve immunization?<br>• Can you tell me more about that?
**I'd like to understand the process you follow to immunize a child, starting from the very beginning.**<br><br>**Probe**:<br>• Does it involve work for you even before the family arrives at the centre for vaccination?<br>• Can you summarize the procedure of immunization in around five steps, starting once a family arrives at the centre for vaccination? [Note: Adjust this question for non-clinic settings if required.]<br>• Are there follow-ups or steps involved once they leave the centre? [Note: Other probes, such as ongoing door to door, systems of recording vaccinations, making vaccination cards and so on, could be added as required.
**What do you find works in helping families stay up to date with immunization?**<br><br>**Probe**:<br>• What helps them to attend appointments? [Note: This is to probe for practical issues.]<br>• What helps those who are hesitant about getting their children vaccinated?
**What do you find difficult when it comes to helping families stay up to date with immunization?**<br><br>**Probe**:<br>• Which part of the process you described before do you find the hardest to complete? Why is that?<br>• Can you give some examples of reasons people give when their child has fallen behind the vaccination schedule?<br>• Can you give some examples of reasons that people give for refusing vaccines for their children?
**If you had the chance, what would you do to improve immunization services in your area?**

Adapted BeSD Tool - Qualitative Questions for Program Managers
Solomon Islands - March 2024

**Section 1: Confirming consent and eligibility**	
Document Date of interview	DATE
Confirm consent has been provided	YES<br>NO
Confirm participant meets eligibility criteria:<br>*In a position of management or supervision of an immunisation or EPI program*	YES<br>NO

**Record Study Identification Number**	
Location of Interview	*List name of*<br>VILLAGE<br>WARD/ZONE<br>DISTRICT
Hello, I am [INTERVIEWER'S NAME] with [INSTITUTION OR ORGANIZATION NAME].<br>We are interviewing people to help improve vaccination services in [NAME OF COUNTRY].<br>The interview is expected to take __ minutes.<br>Your participation is completely voluntary and anonymous. The answers you give will be completely confidential. If you do not want to answer a question or wish to stop the interview, just let me know. Would you be willing to take part in an interview with me?<br>[If audio recording the interview]<br>Would you be happy for me to record our conversation?	
What is your gender?	Woman<br>Man<br>Other<br>Prefer not to say?
How old is the participant in years?	Less than 20 years<br>20 – 29 years<br>30 – 39 years<br>40 – 49 years<br>50 – 59 years<br>60 years or above
What is the highest level of education you have completed?	None<br>Primary (grade 8 or less)<br>High (grade 9, or 10)<br>Secondary (grade 11 or 12)<br>Tertiary college<br>Other (please specify)
Qualification	Doctor<br>Nurse<br>Public Health Practitioner

	Other (please specify)
How long have you been working in this profession?	<5 years<br>5-<10 years<br>≥10 years

Question
**Tell me a little about yourself and your current role.**<br><br>**Probe:**<br>• To what extent does your work involve childhood immunization?<br>• What kinds of immunization-related activities are you responsible for (e.g., surveillance, campaigns, communications)?<br>• Can you tell me more about those?
**What makes the provision of childhood immunization a success in your area?**<br><br>**Probe:**<br>• Are there specific examples you can describe?
**What do you think are the difficulties when it comes to providing childhood immunization in your area?**<br><br>**Probe:**<br>• Do you face difficulties with children falling behind the vaccination schedule in your area?<br>• Can you describe those difficulties?<br>• Do you face difficulties with parents refusing vaccines for their children?<br>• Are you able to overcome these challenges? How?
**If you had the chance, what would you do to improve the childhood immunization situation in your area?**
